# Understanding poor sleep in emerging adulthood: the role of pre-sleep cognitive processes in daily life

**DOI:** 10.1093/sleepadvances/zpag057

**Published:** 2026-06-05

**Authors:** Alexander Ariu, Andrea H Meyer, Simone Munsch, Nadine Messerli-Bürgy

**Affiliations:** Family and Development Research Center, Institute of Psychology, University of Lausanne, Quartier Mouline, Bâtiment Géopolis, 1015 Lausanne, Vaud, Switzerland; Clinical Psychology and Psychotherapy, Department of Psychology, University of Fribourg, Rue P.-A.-de-Faucigny 2, 1700 Fribourg, Fribourg, Switzerland; Clinical Psychology and Epidemiology, Faculty of Psychology, University of Basel, Missionsstrasse 62A, 4055 Basel, Basel, Switzerland; Clinical Psychology and Psychotherapy, Department of Psychology, University of Fribourg, Rue P.-A.-de-Faucigny 2, 1700 Fribourg, Fribourg, Switzerland; Food Research and Innovation Center, Cluster Food and Mental Health/Psychology University of Fribourg, Chemin du Musée 9, 1700 Fribourg, Fribourg, Switzerland; Family and Development Research Center, Institute of Psychology, University of Lausanne, Quartier Mouline, Bâtiment Géopolis, 1015 Lausanne, Vaud, Switzerland

**Keywords:** pre-sleep cognitive processes, sleep quality, ecological momentary assessment, emerging adulthood

## Abstract

**Study Objectives:**

Theoretical perspectives suggest that pre-sleep cognitive processes, including cognitive arousal, rumination, and stress can impair sleep. However, previous studies have relied on cross-sectional designs, limiting insight into how these cognitive processes relate to sleep on a night-to-night basis. In addition, anticipatory stress has received little empirical attention, despite its relevance for poor sleep. To address these gaps, the present study examined the associations of pre-sleep cognitive arousal, pre-sleep rumination, and anticipatory stress with sleep quality, sleep duration, and sleep onset latency both when averaged across 7 days (between-person level) and on a day-to-day basis (within-person level).

**Methods:**

A total of 166 emerging adults (*M_age_* = 20.36 years, 80.1% female) completed smartphone-based surveys during 7 consecutive days, reporting on pre-sleep cognitive processes (cognitive arousal, rumination, anticipatory stress) each evening and sleep outcomes each morning.

**Results:**

Linear mixed-effects models showed that, at the between-person level, higher average levels of all three pre-sleep cognitive processes across the week were associated with poorer sleep quality and shorter sleep duration. No significant associations were found for sleep onset latency. At the within-person level, all three pre-sleep cognitive processes were not related to next-morning sleep outcomes.

**Conclusions:**

Overall, the findings suggest that stable individual differences in pre-sleep cognitive processes, rather than short-term changes, are linked to sleep quality and duration in emerging adults.

**Statement of Significance**

To clarify whether pre-sleep cognitive arousal, pre-sleep rumination, and anticipatory stress show both stable (trait-like) and night-to-night (state) variation, we examined how these pre-sleep cognitive processes relate to sleep in emerging adults. Our findings suggest that trait-like levels of all three cognitive processes are associated with poorer sleep quality and shorter sleep duration, while sleep onset latency appears unaffected. In contrast, short-term nightly changes in these processes were not related to sleep outcomes. Overall, stable pre-sleep cognitive tendencies may be more relevant for sleep than dynamic nightly fluctuations in emerging adults, with important implications for prevention and therapeutic programs.

## Graphical Abstract

**Graphical Abstract:**
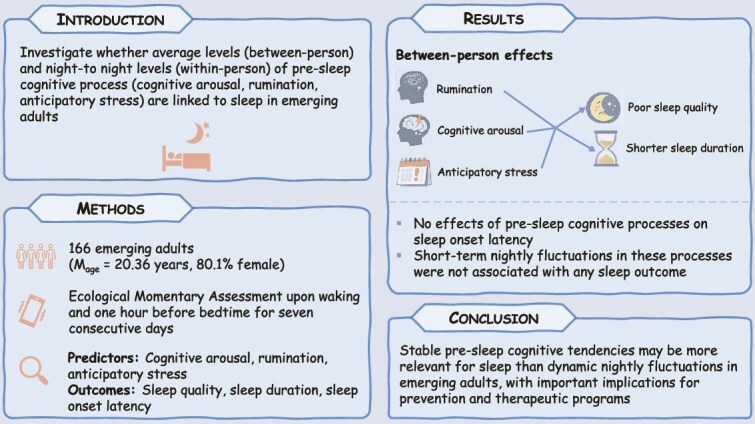

## Introduction

Sleep quality refers to a subjective evaluation of one’s overall sleep experience, including both nighttime aspects (e.g. sleep latency, sleep duration, sleep efficiency, awakenings, restfulness) and daytime consequences such as tiredness or alertness [1, 2]. Poor sleep quality is particularly problematic during emerging adulthood (aged 18–26 years) [3, 4], as prevalence rates are especially high in this age group. International estimates suggest that 61% to 66% of emerging adults report poor sleep quality [5–7], and recent Swiss data indicate that 49% of young adults aged 18 to 35 reported insufficient or non-restorative sleep in the past 12 months [8]. Moreover, poor sleep represents a transdiagnostic risk factor for various psychopathologies such as depression or anxiety in emerging adults [9, 10]. Therefore, it is important to investigate the factors that contribute to poor sleep quality.

Several etiological models have been proposed to explain the onset and maintenance of insomnia, including the Cognitive Model of Insomnia [11] and the Hyperarousal Model of Insomnia [12]. A common aspect of these models is the assumption that various forms of arousal during the pre-sleep period play a central role in insomnia. In this context, pre-sleep arousal is defined as a heightened state of activation prior to sleep that encompasses both somatic (e.g. heart racing, muscle tension) and cognitive aspects (e.g. racing thoughts, worry) [13]. Empirical work consistently shows that individuals with insomnia report higher levels of pre-sleep cognitive arousal than good sleepers (e.g. [14–16]). Besides this, elevated pre-sleep cognitive arousal has also been observed in healthy individuals, suggesting that such activation may represent an early vulnerability factor for the development of sleep disturbances (e.g. [15, 17]). It is further assumed that pre-sleep cognitive arousal contributes to emotional distress, and that increased emotional reactivity mediates the link between cognitive and somatic pre-sleep arousal [11, 18]. Therefore, when individuals experience difficulties regulating their emotions, pre-sleep cognitive arousal may intensify further, creating a self-reinforcing cycle of negative thoughts and wakefulness [19, 20]. From a dynamic perspective, differences in average pre-sleep cognitive arousal may reflect a person’s habitual tendency to enter the night in a heightened cognitive state, whereas deviations from this personal average may capture short-term elevations that can trigger sleep disruption on a given night. This distinction is important because it helps disentangle trait-like vulnerability from nightly fluctuations in pre-sleep cognitive arousal.

Another component of the Cognitive Model of Insomnia is rumination [11]. Depending on the literature, rumination is conceptualized either as an emotion regulation strategy or as a form of perseverative cognition [21, 22], both of which refer to repetitive, stress-related negative thinking about past experiences or current negative feelings [22, 23]. In a meta-analysis of 32 non-clinical studies, Clancy and colleagues found small effects of rumination on sleep onset latency and sleep duration and medium-sized effects on sleep quality [24]. However, this evidence is largely based on cross-sectional data, and only a few studies have used experimental designs (e.g. [25]) or diary approaches to assess pre-sleep rumination on a given night (e.g. [26]). As a result, research on daily pre-sleep rumination in relation to sleep is still limited, and currently available data on multi-day diary studies have produced mixed findings. A 7-day study of emerging adults with depressive symptoms showed that higher nightly pre-sleep rumination predicted longer subjective sleep onset latency [27], whereas other daily diary studies spanning 8 to 14 days reported weaker and less consistent associations with sleep onset latency, duration, and quality [28, 29]. Moreover, in those studies, measurement time points were limited to single evening assessment [28, 29] or following morning measures asking participants to retrospectively estimate their rumination level [27] and did not focus on the pre-sleep period.

In addition to rumination and arousal, stressors play an important role in the Hyperarousal Model of Insomnia [12]. Stressors are particularly prevalent among emerging adults, as this population is often exposed to considerable instability [30, 31]. It is therefore not surprising that stress and insomnia are moderately associated in a meta-analysis of emerging adults [31]. However, other stress-related constructs, such as anticipatory stress, have not yet been explicitly addressed in the two insomnia models described above. Anticipatory stress refers to the experience of feeling stressed or worried in the evening about events or demands anticipated for the following day and can be conceptualized as a form of perseverative cognition [22]. Anticipatory stress might contribute to the development and maintenance of sleep disturbances [32, 33], and experimental nap research revealed that merely anticipatory social-evaluative stress can be sufficient to impair objective indicators of sleep, including longer sleep onset latency and reduced slow-wave sleep [34]. However, it remains unclear whether anticipatory stress is mainly a stable characteristic, such that individuals with higher average levels tend to experience poorer sleep, or whether deviations from this personal average reflect short-term elevations that can interfere with sleep on a given night.

Although some studies have investigated nightly variations in pre-sleep cognitive arousal, pre-sleep rumination, and stress in relation to sleep among emerging adults (e.g. [27, 35, 36]), anticipatory stress has not been the focus of research so far, although it is known to impact mental health [37]. Furthermore, the interplay of these factors in daily life has rarely been examined together. Moreover, it remains unclear whether their impact on sleep reflects more stable or dynamic night-to-night fluctuations in emerging adults. Therefore, repeated assessments close to bedtime are needed to clarify their dynamic associations with subsequent sleep in a student population. To address these gaps, the present study used smartphone-based ecological momentary assessment (EMA) to assess pre-sleep cognitive arousal, pre-sleep rumination, and anticipatory stress in the evening during 7 consecutive days. We hypothesized that higher levels in these characteristics are associated with longer sleep onset latency, shorter sleep duration, and poorer sleep quality when averaged across a 7-day period (between-person level) as well as on a day-to-day basis (within-person level).

## Materials and methods

### Participants

A total of 166 first-year psychology students were recruited from two Swiss universities (Lausanne and Fribourg), representing both French- and German-speaking regions. Recruitment took place between November 2024 and March 2025 through in-class announcements and campus advertisements. Participation was voluntary and compensated with course credits in line with university guidelines. Eligible participants had to be between 18 and 24 years, fluent in French or German, own a smartphone with a Swiss phone number, have daily internet access, and stay in Switzerland throughout the 8-day study period. Students were excluded if they reported atypical sleep patterns in the prior week (e.g. due to jet lag or shift work).

### Study design

The study employed a repeated-measures design to examine both between-person differences (i.e. how individuals’ 7-day average levels of pre-sleep characteristics relate to their 7-day average levels of sleep outcomes) and within-person fluctuations (i.e. how daily fluctuations in pre-sleep characteristics relate to subsequent nightly fluctuations in sleep outcomes). Data were collected over 8 consecutive days using Research Electronic Data Capture (REDCap). On day 1, participants completed a baseline questionnaire at home on their computer. From day 2 to 8, they received four daily SMS prompts linking to brief online surveys (7–8 minutes each): upon waking, at midday, at 4 p.m., and approximately 1 hour before bedtime. The present analyses focus on the morning and evening assessments covering sleep and pre-sleep cognitive processes. Data from the midday and afternoon surveys assessing other cognitive and emotional processes are reported elsewhere. To improve adherence, prompt times were tailored to each participant’s routine (e.g. wake-up time and bedtime). If one of the surveys was not completed by the participant, up to two automated reminders were sent at 45-minute intervals, and surveys not completed within 2 hours of the initial prompt were coded as missing.

### Procedure

After enrollment, participants received a REDCap link with detailed study information and provided written informed consent, followed by access to an online questionnaire assessing sociodemographic characteristics and general sleep-related measures (e.g. sleep patterns, sleep reactivity, and pre-sleep arousal). Subsequently, participants were instructed about the EMA procedure, including prompt timing, response instructions, and reminders. Across the study, participants could complete up to 14 brief morning and evening surveys. The study protocol was approved by the Research Ethics Commission of the University of Lausanne (CER-UNIL) on September 27, 2024 (Project no. E_SSP_042024_00001), adhered to the Declaration of Helsinki, and was preregistered on the Open Science Framework (https://osf.io/e3x7w) on March 5, 2025.

### Measures

#### Sociodemographics

Participants reported age, gender identity, language, and migration background. Subjective social status was measured with the MacArthur Subjective Social Status ladder [38], rated from 1 (*lowest*) to 10 (*highest*).

#### Upcoming exams

To assess proximity to upcoming exams, participants were asked, “In which month and year will you take your next exam?”, allowing estimation of the time between study participation and the next exam.

#### Subjective sleep quality

The Pittsburgh Sleep Quality Index (PSQI) [1] is a 19-item self-report questionnaire assessing subjective sleep quality over the previous month. It comprises seven sleep components: subjective sleep quality, sleep onset latency, sleep duration, habitual sleep efficiency, sleep disturbances, use of sleeping medication, and daytime dysfunction. Each component is scored on a scale from 0 to 3, with a total score ranging from 0 (*no sleep difficulties*) to 21 (*severe sleep difficulties in all domains*). A global score above 5 indicates poor subjective sleep quality [1]. In this sample, internal consistency was modest (α = .65), which is lower than in other studies (α = .83–.87) [1, 39].

#### Sleep reactivity

The Ford Insomnia Response to Stress Test (FIRST) [40] is a nine-item self-report measure of trait sleep reactivity, which is an individual’s predisposition to experience sleep disturbances in response to stress. Responses are given on a 4-point Likert scale ranging from 1 (*not likely*) to 4 (*very likely*), with a total score between 9 and 36. Higher scores indicate greater sleep reactivity, with scores above 16 suggesting moderate and scores above 18 high sleep reactivity [41]. Internal consistency in this sample was good (⍺ = .82) and comparable to previous research (α = .83) [40].

#### Pre-sleep arousal

The Pre-Sleep Arousal Scale (PSAS) [13] is a self-report instrument measuring general pre-sleep arousal across two dimensions: pre-sleep cognitive and pre-sleep somatic arousal. It consists of 16 items, with 8 items per subscale, rated on a 5-point Likert scale, ranging from 1 (*not at all*) to 5 (*extremely*). Subscale scores range from 8 to 40, with higher scores indicating greater pre-sleep arousal. In this sample, internal consistency was high for pre-sleep cognitive arousal (α = .89) and acceptable for pre-sleep somatic arousal (α = .75), consistent with previous research in college students (α = .88 for cognitive, α = .79 for somatic) [13].

#### Daily morning and evening assessments

Each morning during the 7-day EMA period, participants reported on the previous night’s sleep using adapted items from the brief PSQI [42]. This included sleep onset latency (1 item), indicating how many minutes it took them to fall asleep, sleep duration (2 items), calculated as the time difference between self-reported bedtime and wake-up time, and subjective sleep quality (1 item), reflecting how rested and refreshed participants felt upon awakening.

Evening surveys focused on pre-sleep arousal, pre-sleep rumination, and anticipatory stress. For arousal and rumination, we selected items with the highest factor loadings from the original validation studies. Pre-sleep cognitive arousal was assessed with five items from the cognitive subscale of the PSAS [13], and pre-sleep rumination with five items adapted from the Brief State Rumination Inventory [43]. For each scale, average levels were calculated. Both constructs showed excellent between-person and good within-person reliability (cognitive arousal: α = .94 between-person, α = .84 within-person; rumination: α = .97 between-person, α = .87 within-person). Anticipatory stress was assessed with a single item adapted from Block and colleagues [44] for this project (“I am experiencing a stressful day tomorrow”), rated on a visual analog scale from 0 (*not at all stressful*) to 100 (*very stressful*). All EMA items and response formats are provided in the supplementary material.

### Statistical analysis

Linear mixed-effects models (LMMs) were conducted using R (version 4.4.1) [45] with the *nlme* package to account for repeated daily assessments nested within individuals [46]. LMMs accommodate within-person dependencies and missing data, allowing all available observations to be retained [47]. Models tested between-person (person-mean) and within-person (night-to-night) associations between the predictors (pre-sleep cognitive arousal, pre-sleep rumination, and anticipatory stress) and the outcomes (sleep onset latency, sleep duration, and sleep quality). Between-person effects were estimated by including each participant’s mean predictor value during the 7-day EMA period (person-mean). Within-person effects were modeled by centering daily predictors around each participant’s mean (centering within clusters), thereby avoiding conflation between within- and between-person effects [48]. Random-intercept models were used, as random slopes generally did not improve fit in any model. For each sleep outcome, three separate LMMs were estimated, each including one predictor (pre-sleep cognitive arousal, pre-sleep rumination, or anticipatory stress) with both its between- and within-person components. In addition to the basic models, adjusted models included study day, study site (Lausanne vs. Fribourg), age, and gender as covariates to account for potential time-related changes, site differences, and demographic characteristics. Gender was dummy-coded for female and male participants.

Prior to analysis, model assumptions were evaluated using visual diagnostics of residuals and random intercept coefficients (histograms, boxplots, Q–Q plots, homoscedasticity), implemented with *HLMdiag* [49]. Overall, the diagnostics indicated normal distributions and no major violations of homoscedasticity. Sleep onset latency was the only exception, as residuals deviated from normality, so the outcome was log-transformed using log(1 + x), which resulted in acceptable residuals and homoscedasticity. Statistical significance was evaluated with an alpha level of .05, and *p*-values were adjusted for multiple testing using the Benjamini–Hochberg false discovery rate procedure [50], applied within each outcome across the three models that included all predictors. LMMs were estimated using maximum likelihood [51] and explained variance was assessed using marginal *R^2^* values for fixed effects in LMMs [52], computed with the *r2glmm* package [53]. For descriptive purposes, intraclass correlation coefficients (ICCs) were calculated for all outcomes and predictors in each model, using the *performance* package [54]. ICCs were used to indicate the degree to which each construct reflected stable between-person differences versus within-person fluctuations, with values around .00 to .40 in EMA research typically suggesting that most variance is within-person [47].

## Results

### Sample characteristics

Table 1 presents descriptive statistics for all sample and general sleep characteristics (e.g. sleep patterns, sleep reactivity, and pre-sleep arousal). Results show that subjective social status was modest in our sample, and comparable to previous research (*M_Range_*: 5.6–6.1) [55]. Regarding upcoming exams, 9 participants (5.4%) reported that their next exam would take place in the same month, whereas 64 participants (38.5%) reported that it would take place in the following month.

**Table 1:** Descriptive statistics for sample characteristics, sleep variables, and pre-sleep cognitive processes

Variable	*n* (%) / *M* (*SD*)	Range
Age, *M* (*SD*)	20.36 (1.45)	18–24 years
Gender, *n* (%)		
Female	133 (80.1)	
Male	26 (15.7)	
Non-binary/genderfluid	4 (2.4)	
Unsure	2 (1.2)	
No response	1 (0.6)	
Native language, *n* (%)		
French	141 (84.9)	
German	25 (15.1)	
Migration background, *n* (%)		
Yes	102 (61.4)	
No	64 (38.6)	
Subjective social status, *M* (*SD*)	5.74 (1.45)	3–8
Subjective sleep quality (PSQI)		
Sleep quality (Component 1), *n* (%)		
Very good	13 (7.8)	
Fairly good	104 (62.7)	
Fairly bad	46 (27.7)	
Very bad	3 (1.8)	
Sleep onset latency in minutes (Component 2), *M* (*SD*)	41.01 (48.77)	2–420
Sleep duration in hours (Component 3), *M* (*SD*)	7.17 (1.14)	3.00–9.53
Total score, *M* (*SD*)	6.73 (3.05)	1–17
Sleep reactivity (FIRST), *M* (*SD*)	23.35 (5.51)	9–35
Pre-sleep arousal (PSAS), *M* (*SD*)		
Pre-sleep cognitive arousal	23.73 (7.76)	8–38
Pre-sleep somatic arousal	14.42 (4.86)	8–32

*Note.* Each variable is described by its number, frequency in %, mean (*M*), standard deviation (*SD*), or range for the total sample of *N* = 166. PSQI = Pittsburgh Sleep Quality Index; FIRST = Ford Insomnia Response to Stress Test; PSAS = Pre-Sleep Arousal Scale.

Based on the PSQI cutoff of >5 for poor subjective sleep quality [1], 102 participants (61.4%) were classified as poor sleepers. However, a closer examination of sleep quality revealed that most participants rated their sleep as “fairly good”, while more than a quarter reported it as “fairly bad”, and only a small number rated their sleep as “very good” or “very bad”. Further, participants reported an average sleep duration that was within the adequate range, but the average level of sleep onset latency indicated a prolonged time to fall asleep (see supplementary Table S1 for more details). Regarding sleep reactivity, most participants (83.7%) showed high levels of vulnerability to stress-induced sleep disturbances. Besides this, the pre-sleep cognitive arousal score was generally higher than the pre-sleep somatic arousal score in this sample.

Across a total of 2324 scheduled morning and evening prompts, 2109 surveys were completed, resulting in an overall completion rate of 90.7%. Most surveys were completed without reminders (63.1%), while 21.9% and 5.7% were completed after one and two reminders, respectively. Completion rates were consistently high across study days (88.2%–93.1%) and across time points, with 89.1% completion in the morning and 92.3% in the evening. On average, participants completed 6.5 of 7 morning surveys and 6.2 of 7 evening surveys, with a range of 1 to 7 completed surveys at both time points.

### Characteristics of study variables

The ICCs for sleep onset latency (17%), sleep duration (25%), and sleep quality (27%) indicated that only a smaller proportion of variance reflected stable between-person differences, whereas the majority was attributable to within-person fluctuations over time (73%–83%). As shown in Table 2, participants reported moderate levels of sleep onset latency, sleep duration, and sleep quality, each accompanied by broad ranges and high standard deviations, indicating substantial variability in responses. Similarly, scores for pre-sleep cognitive arousal, pre-sleep rumination, and anticipatory stress were moderately high and displayed broad ranges.

**Table 2:** Descriptive statistics for EMA measures

Variable	*M* (*SD*)	Range
Sleep onset latency in minutes (*n* = 1038)	31.02 (56.36)	0–480
Sleep duration in hours (*n* = 1037)	7.76 (1.54)	1.77–12.58
Sleep quality (*n* = 1038)	48.11 (23.98)	0–100
Pre-sleep cognitive arousal (*n* = 1074)	1.93 (0.94)	1–5
Pre-sleep rumination (*n* = 1074)	28.22 (27.98)	0–100
Anticipatory stress (*n* = 1074)	34.91 (28.20)	0–100

*Note.* Each variable is described by its mean (*M*), standard deviation (*SD*), and range. *n* = valid observations per variable during all EMA assessments.

The ICCs for the predictors pre-sleep cognitive arousal (54%) and pre-sleep rumination (58%) indicated that a larger proportion of variance was attributable to stable between-person differences, with comparatively less variance reflecting within-person fluctuations (42%–46%). Anticipatory stress showed a lower ICC (37%), suggesting comparatively greater within-person variability (63%).

### Correlations of study variables

At the between-person level, pre-sleep cognitive arousal, pre-sleep rumination, and anticipatory stress were strongly correlated, indicating that individuals who reported higher average levels of one process also tended to report higher average levels of the other processes (see Figure S1). In contrast, within-person correlations were substantially smaller, suggesting weaker associations between daily variations in these processes (see Figure S2). In addition, associations between baseline measures and between-person components were examined to assess convergence between questionnaire-based constructs and person-level means of daily cognitive processes. Baseline cognitive arousal and sleep reactivity showed moderate to high correlations with average levels of daily pre-sleep cognitive arousal, pre-sleep rumination, and anticipatory stress, indicating that higher trait-level vulnerability was reflected in elevated levels of perseverative cognitive processes in daily life (see Figure S3).

### Associations of pre-sleep cognitive processes with sleep outcomes

At the between-person level, individuals who reported higher average levels of pre-sleep cognitive arousal, pre-sleep rumination, and anticipatory stress during the entire week showed significantly lower average sleep quality and sleep duration (see Table 3). No associations were found between any of the three weekly average pre-sleep cognitive process variables and average sleep onset latency. Effect sizes ranged from 0% to 2.9% of explained variance. At the within-person level, no significant associations emerged, indicating that night-to-night fluctuations in pre-sleep cognitive arousal, pre-sleep rumination, and anticipatory stress were not related to daily changes in sleep quality, sleep duration, or sleep onset latency.

**Table 3:** Associations between pre-sleep cognitive processes and sleep outcomes

		Fixed effects			
Model	Predictor	Coefficient (*SE*)	95% CI	*p_FDR_*	*R^2^*
**Sleep quality**					
Pre-sleep cognitive arousal	Pre-sleep cognitive arousal (between)	−5.61 (1.56)	[−8.69, −2.53]	.001^**^	.029
	Pre-sleep cognitive arousal (within)	−0.74 (1.12)	[−2.93, 1.46]	.613	.000
Pre-sleep rumination	Pre-sleep rumination (between)	−1.63 (0.51)	[−2.64, −0.62]	.003^**^	.023
	Pre-sleep rumination (within)	0.13 (0.40)	[−0.66, 0.93]	.739	.000
Anticipatory stress	Anticipatory stress (between)	−2.16 (0.59)	[−3.33, −0.98]	.001^**^	.029
	Anticipatory stress (within)	−0.28 (0.32)	[−0.90, 0.35]	.580	.001
**Sleep duration**					
Pre-sleep cognitive arousal	Pre-sleep cognitive arousal (between)	−2.97 (1.00)	[−4.94, −0.99]	.007^**^	.020
	Pre-sleep cognitive arousal (within)	−1.40 (0.72)	[−2.81, 0.01]	.079	.003
Pre-sleep rumination	Pre-sleep rumination (between)	−1.00 (0.33)	[−1.64, −0.34]	.007^**^	.021
	Pre-sleep rumination (within)	−0.39 (0.26)	[−0.89, 0.12]	.162	.002
Anticipatory stress	Anticipatory stress (between)	−1.16 (0.38)	[−1.92, −0.41]	.007^**^	.020
	Anticipatory stress (within)	0.16 (0.20)	[−0.24, 0.56]	.438	.000
**Sleep onset latency**					
Pre-sleep cognitive arousal	Pre-sleep cognitive arousal (between)	0.55 (0.77)	[−0.96, 2.06]	.880	.001
	Pre-sleep cognitive arousal (within)	0.47 (0.48)	[−0.48, 1.42]	.880	.001
Pre-sleep rumination	Pre-sleep rumination (between)	0.30 (0.25)	[−0.20, 0.79]	.880	.004
	Pre-sleep rumination (within)	0.01 (0.17)	[−0.32, 0.36]	.920	.000
Anticipatory stress	Anticipatory stress (between)	−0.10 (0.29)	[−0.68, 0.48]	.880	.000
	Anticipatory stress (within)	−0.07 (0.14)	[−0.34, 0.20]	.880	.000

*Note*. Between-person associations were modeled using person-level means, and within-person associations were modeled using cluster-mean centering. *SE* = standard error of the coefficient; 95% CI = lower (2.5%) and upper limit (97.5%) of the confidence interval with 95%; *p_FDR_* = Benjamini–Hochberg false discovery rate procedure corrected *p*-value; *R^2^* = Proportion of variance in sleep quality uniquely explained by each fixed effect.

^*^ *p_FDR_* < .05, ^**^p_FDR_ < .01, ^***^p_FDR_ < .001.

In the adjusted models including study day, study site, age, and gender as covariates, the pattern of findings was largely consistent with the main analyses, although the between-person association between pre-sleep rumination and sleep quality no longer reached significance (see Supplementary Table S2 for full model results). Study day, age, and gender were not significantly associated with any sleep outcome, whereas study site was significantly associated with sleep duration, but not with sleep quality or sleep onset latency, indicating shorter sleep duration among participants recruited at the University of Lausanne than among those recruited at the University of Fribourg.

## Discussion

This study examined whether three pre-sleep cognitive processes are associated with night-to-night sleep quality, sleep duration, and sleep onset latency in emerging adults. Consistent with our between-person hypotheses, participants with higher weekly average levels of pre-sleep cognitive arousal, pre-sleep rumination, and anticipatory stress reported poorer average sleep quality and sleep duration. Contrary to our within-person hypotheses, nightly fluctuations in these pre-sleep processes were not related to next-morning sleep outcomes. Moreover, none of the pre-sleep processes were associated with sleep onset latency at either the between-person or within-person level.

These findings are particularly relevant given the high sleep vulnerability in this sample. Overall, 61.4% of participants met the cutoff for poor sleep quality and 83.7% showed high sleep reactivity, consistent with previous estimates in student populations [5–7], and the average sleep reactivity was higher than that reported in the original validation study [40]. Nevertheless, sleep outcomes showed substantial within-person variability, with 68%–78% of variance reflecting night-to-night fluctuations. Despite this variance, nightly changes in pre-sleep cognitive arousal, pre-sleep rumination, and anticipatory stress were not associated with next morning sleep changes. One explanation may be the limited within-person variability of pre-sleep cognitive arousal and pre-sleep rumination, as reflected in their ICCs reported above, which may partly be related to the study context. Stronger within-person fluctuations in these processes might have been more likely during a period in which stress levels varied more markedly, such as an exam period within the academic semester. In our study, however, only 5.4% of participants reported that their next exam would take place in the same month, and 38.5% in the following month, suggesting that the majority were not participating during a period of more immediate academic stress. Thus, only a small proportion of the sample was likely facing a more immediate academic stressor. It is therefore possible that daily fluctuations in pre-sleep cognitive processes were too small to meaningfully influence sleep from one night to the next, suggesting that these processes may be more relevant as relatively stable vulnerability factors under low stress conditions.

Our findings showed that higher weekly average levels of pre-sleep cognitive arousal were associated with poorer sleep quality and shorter sleep duration, whereas no between-person association emerged for sleep onset latency, and no significant within-person associations were observed for any sleep outcome. Notably, the mean pre-sleep cognitive arousal score on the PSAS in our sample (see Table 1) was nearly twice as high as values reported in earlier research [13], but comparable to levels reported in 18- to 25-year-olds [56]. However, evidence on between-person associations between pre-sleep cognitive arousal and sleep in daily-life studies is limited. To our knowledge, only Winzeler and colleagues [36] examined this and found no association with sleep quality, which contrasts with our results. Our within-person findings contrast with prior mediation studies reporting within-person pathways from pre-sleep cognitive arousal to subjective sleep quality [35, 36]. Specifically, both 14-day daily diary studies found that on days when individuals experienced higher pre-sleep cognitive arousal than usual, they subsequently reported poorer subjective sleep quality. Several methodological differences may help explain these discrepancies. First, both studies assessed pre-sleep cognitive arousal every evening using the full PSAS [13], whereas the current study relied on five adapted items used in brief evening surveys. Second, both studies examined these associations within mediation frameworks, whereas no mediation was conducted in the present study.

Our findings on pre-sleep rumination align with previous research. At the between-person level, higher weekly average rumination was associated with poorer sleep quality and shorter sleep duration, but not with sleep onset latency. In contrast, Slavish and colleagues [29] found no such between-person association in their 14-day daily diary study for sleep quality and sleep onset latency, likely due to their broader assessment of rumination across the entire day, which did not specifically capture pre-sleep thought patterns in a comparable way, but results might perhaps differ also because of the age difference of the samples. Their sample consisted of middle-aged adults, who ruminate less than younger ones [57, 58]. At the within-person level, our results are consistent with some of the previous studies showing no association between nightly fluctuations in pre-sleep rumination with sleep quality, sleep duration, and sleep onset latency [28, 29]. However, Pillai and colleagues [27] found a positive association between day-to-day pre-sleep rumination and sleep onset latency in participants with elevated depressive symptoms, a condition that represents chronic stress and can amplify the link between rumination and delayed sleep [59]. A similar association was found by Van Laethem and colleagues [60] between increased nightly perseverative cognition and poorer subjective sleep quality but not sleep duration or sleep onset latency during a specific context, as data were collected around PhD defenses when stress and cognitive arousal are likely elevated. Therefore, we assume that nightly fluctuations in pre-sleep rumination become relevant for sleep under elevated stress.

For anticipatory stress, higher weekly average levels were associated with poorer sleep quality and shorter sleep duration, but not with sleep onset latency. These findings are in line with previous studies showing similar associations of anticipatory stress for sleep quality and sleep duration in middle-aged adults [44]. At the within-person level, anticipatory stress was not related to any sleep outcome, contrasting with Åkerstedt and colleagues [61], who found that bedtime stress and worries predicted poorer subjective sleep quality, possibly because their 42-day design captured more intraindividual variability than our 7-day assessment. Other studies focused on momentary evening stress rather than anticipatory stress and found no direct associations with sleep outcomes [28, 60]. Instead, stress effects on sleep were mediated by rumination, which predicted poorer subjective sleep quality, more nighttime wakefulness, and reduced sleep efficiency [28, 60].

Our study has several strengths. The daily EMA design assessed pre-sleep cognitive processes each evening and sleep outcomes the following morning, enabling the estimation of both between- and within-person effects. In addition, participant compliance was high, and the analyses were sufficiently powered for linear mixed-effects modeling [62] and met the preregistered large sample size (https://osf.io/e3x7w). As an additional strength, our adjusted models, including study day, study site, age, and gender as covariates, showed that the overall pattern of findings was largely stable after adjustment. This supports the robustness of the main results. Only the between-person association between pre-sleep rumination and sleep quality no longer reached significance, indicating that this specific finding should be interpreted with some caution. However, some limitations must be considered. First, the study was conducted in a sample of psychology students recruited at the Universities of Lausanne and Fribourg, in which women were overrepresented (80.1%), which limits the generalizability of the findings to the broader population of emerging adults. However, this gender distribution reflects the recruitment pool, given that women account for 74.1% of Bachelor’s students in the humanities and social sciences in Switzerland [63]. Therefore, the findings may not generalize to non-student populations or to samples characterized by greater demographic diversity, particularly with regard to gender. Future work should therefore replicate this EMA approach in a broader population and also include participants with more severe sleep disturbances, such as insomnia, in whom pre-sleep cognitive processes may be more pronounced [16, 64]. Second, between-person predictors were computed as person means across a short 7-day period, which can introduce sampling error and underestimate between-person effects known as Lüdtke’s bias [65]. Therefore, future studies should employ longer sampling periods to obtain more reliable person-level aggregates. Third, the internal consistency of the sleep quality questionnaire (PSQI) in this sample was modest, likely because the sleep medication component contributed little variance, as most participants did not report sleep problems requiring medication. Notably, this reliability estimate is comparable to healthy participants of other European student samples [66, 67].

Future research should incorporate objective sleep measures such as actigraphy to complement self-reports and provide a more comprehensive understanding of sleep duration and sleep onset latency. In addition, collecting more detailed qualitative information on the content of pre-sleep rumination may help identify whether certain types of concerns, such as work/education-related versus personal life-related thoughts, are particularly relevant for sleep disturbances [60, 68]. Moreover, future studies should assess these processes during periods of heightened acute stress, such as exam phases, as the present study was not specifically conducted in such a context. This may help clarify whether daily associations between pre-sleep cognitive processes and sleep variables become more pronounced under conditions of stronger situational stress. Future studies might further investigate whether associations between pre-sleep cognitive processes and sleep differ between weekdays and weekends. This may be particularly relevant in emerging adults, as prior cross-sectional research suggests that sleep patterns can vary across the week in this age group [69]. In the present study, assessments were conducted across only one week, including 5 weekdays and two weekend days, which limited the possibility of examining such differences in a more robust and differentiated way.

Our findings may be of practical relevance in relation to the link of emotion regulation and sleep. Although standard cognitive-behavioral therapy for insomnia targets dysfunctional beliefs about sleep and sleep restriction so far, this study emphasizes again the need of integrating emotion regulation strategies [70]. Mindfulness-based approaches might complement such treatments [71]. In this line, Sommerhoff and colleagues [72] conducted a 10-day randomized controlled EMA study using a healthy sample of students. They found that practicing a brief 5-minute body scan exercise before sleep was associated with greater reductions in repetitive negative thoughts after 10 days and lower stress levels compared to controls.

In conclusion, individuals who reported higher average levels of pre-sleep cognitive processes experienced poorer subjective sleep quality and shorter sleep duration, suggesting that trait-like pre-sleep cognitive processes may be more relevant for sleep impairments than transient nightly fluctuations in emerging adults. EMA may therefore be a useful tool for future intervention research in clinical populations, as repeated daily assessments could capture how changes in pre-sleep cognitive processes relate to sleep over time and help inform more personalized and adaptive approaches to promoting healthy sleep, particularly in populations at increased risk for sleep problems.

## Supplementary Material

Supplementary_material_SLEEPAdvances_zpag057

## Data Availability

Data will be made available on request.
