# Supplementary material for: Understanding poor sleep in emerging adulthood: the role of pre-sleep cognitive processes in daily life
Source: Sleep Adv. 2026 Jun 5;7(3):zpag057. doi: 10.1093/sleepadvances/zpag057 (PMC13356899; doi:10.1093/sleepadvances/zpag057)
Supplement: Supplementary_material_SLEEPAdvances_zpag057 [file supplementary_material_sleepadvances_zpag057.docx]

**Supplementary material**

**Manuscript title:** Understanding poor sleep in emerging adulthood: The role of pre-sleep cognitive processes in daily life

Alexander Ariu ^1^, Andrea H. Meyer ^2, 3^, Simone Munsch ^2, 4^, Nadine Messerli-Bürgy ^1^

^1^ Family and Development Research Center, Institute of Psychology, University of Lausanne, Quartier Mouline, Bâtiment Géopolis, 1015 Lausanne, Switzerland, [alexander.ariu@unil.ch](mailto:alexander.ariu@unil.ch), [nadine.messerli-burgy@unil.ch](mailto:nadine.messerli-burgy@unil.ch)

^2^ Clinical Psychology and Psychotherapy, Department of Psychology, University of Fribourg, Rue P.-A.-de-Faucigny 2, 1700 Fribourg, Switzerland, andrea.meyer@unifr.ch, [simone.munsch@unifr.ch](mailto:simone.munsch@unifr.ch)

^3^ Clinical Psychology and Epidemiology, Faculty of Psychology, University of Basel, Missionsstrasse 62A, 4055 Basel, Switzerland, [andrea.meyer@unibas.ch](mailto:andrea.meyer@unibas.ch)

^4^ Food Research and Innovation Center (FRIC), Cluster Food and Mental Health / Psychology University of Fribourg, Chemin du Musée 9, 1700 Fribourg, Switzerland, simone.munsch@unifr.ch

**Corresponding author**: Mr. Alexander Ariu, Family and Development Research Center, Institute of Psychology, University of Lausanne, Quartier UNIL-Mouline, Bâtiment Géopolis, 1015 Lausanne, Switzerland. Email: [alexander.ariu@unil.ch](mailto:alexander.ariu@unil.ch)

**Ecological Momentary Assessment: Items and response formats**

**Assessment in the morning upon waking (before school/work)**

| ***Sleep quality***   - How rested or refreshed did you feel when you woke up for the day? [VAS scale: 0= Not at all rested or refreshed; 50 = moderately rested or refreshed; 100 = very rested or refreshed]   ***Sleep duration***   - **Bedtime:** When did you close your eyes to fall asleep? [Specify answer as time window] - **Wake-up time:** What time did you wake up this morning? [Specify answer as time window]   ***Sleep onset latency***   - How long did it take you to fall asleep? [Duration in minutes to fall asleep specified in a time window] |
| --- |

**Assessment in the evening, one hour before bedtime**

| ***Pre-sleep cognitive arousal***  Selected strongest item loading for pre-sleep cognitive arousal of the Pre-Sleep Arousal Scale (Nicassio et al., 1985)   - Right now, I review or ponder the events of the day. [1 = not at all; 2 = slightly; 3 = moderately; 4 = a lot; 5 = extremely] - Right now, I can’t shut off my thoughts. [1 = not at all; 2 = slightly; 3 = moderately; 4 = a lot; 5 = extremely] - Right now, thoughts keep racing through my head. [1 = not at all; 2 = slightly; 3 = moderately; 4 = a lot; 5 = extremely] - Right now, I have depressive or anxious thoughts. [1 = not at all; 2 = slightly; 3 = moderately; 4 = a lot; 5 = extremely] - Right now, I worry about problems other than sleep. [1 = not at all; 2 = slightly; 3 = moderately; 4 = a lot; 5 = extremely]   ***Pre-sleep rumination***  Selected strongest item loading for pre-sleep rumination of the Brief State Rumination Inventory (Marchetti et al., 2018)   - Right now, I wonder why I always feel the way I do. [VAS scale: ranging from “completely disagree” (0) to “completely agree” (100)] - Right now, I am thinking: “why do I have problems other people don’t have?” [VAS scale: ranging from “completely disagree” (0) to “completely agree” (100)] - Right now, I am thinking: “why can’t I handle things better?” [VAS scale: ranging from “completely disagree” (0) to “completely agree” (100)] - Right now, it is hard for me to shut off negative thoughts about myself. [VAS scale: ranging from “completely disagree” (0) to “completely agree” (100)] - Right now, I wonder why I can’t respond in a better way. [VAS scale: ranging from “completely disagree” (0) to “completely agree” (100)]   ***Anticipatory stress***   - I am experiencing a stressful day tomorrow. [0 = not at all stressful; 100 = very stressful] |
| --- |

**Table S1**

*Descriptive statistics for the seven PSQI components and total score*

| **Variable** | ***n* (%) / *M* (*SD*)** | **Range** |
| --- | --- | --- |
| Subjective sleep quality (PSQI) |  |  |
| Sleep quality (Component 1), *n* (%) |  |  |
| Very good | 13 (7.83) |  |
| Fairly good | 104 (62.65) |  |
| Fairly bad | 46 (27.71) |  |
| Very bad | 3 (1.81) |  |
| Sleep onset latency in minutes (Component 2), *M* (*SD*) | 41.01 (48.77) | 2–420 |
| Sleep duration in hours (Component 3), *M* (*SD*) | 7.17 (1.14) | 3.00–9.53 |
| Sleep efficiency in % (Component 4), *M* (*SD*) | 86.30 (12.87) | 35.74–100 |
| Sleep disturbances (Component 5), *n* (%) |  |  |
| Not during the past month | 3 (1.81) |  |
| Less than once a week | 134 (80.72) |  |
| Once or twice a week | 29 (17.47) |  |
| Three or more times a week | - |  |
| Use of sleep medication (Component 6), *n* (%) |  |  |
| Not during the past month | 140 (84.33) |  |
| Less than once a week | 11 (6.63) |  |
| Once or twice a week | 4 (2.41) |  |
| Three or more times a week | 11 (6.63) |  |
| Daytime dysfunction (Component 7), *n* (%) |  |  |
| Not during the past month | 76 (45.78) |  |
| Less than once a week | 48 (28.92) |  |
| Once or twice a week | 36 (21.69) |  |
| Three or more times a week | 6 (3.61) |  |
| Total score, *M* (*SD*) | 6.73 (3.05) | 1–17 |

*Note.* Each variable is described by its number, frequency in %, mean (*M*), standard deviation (*SD*), or range for the total sample of *N* = 166. PSQI = Pittsburgh Sleep Quality Index.

**Figure S1**

*Between-person associations among pre-sleep cognitive arousal, pre-sleep rumination, and anticipatory stress*

*
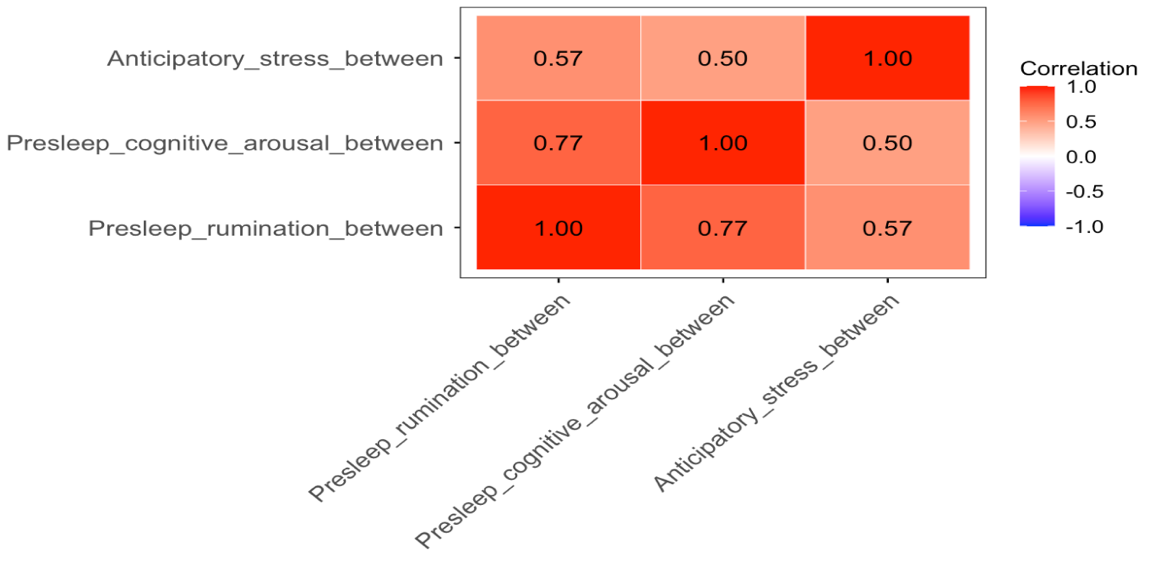
*

*Note*. Heatmap shows between-person Pearson correlations among pre-sleep cognitive arousal, pre-sleep rumination, and anticipatory stress.

**Figure S2**

*Within-person associations among pre-sleep cognitive arousal, pre-sleep rumination, and anticipatory stress*


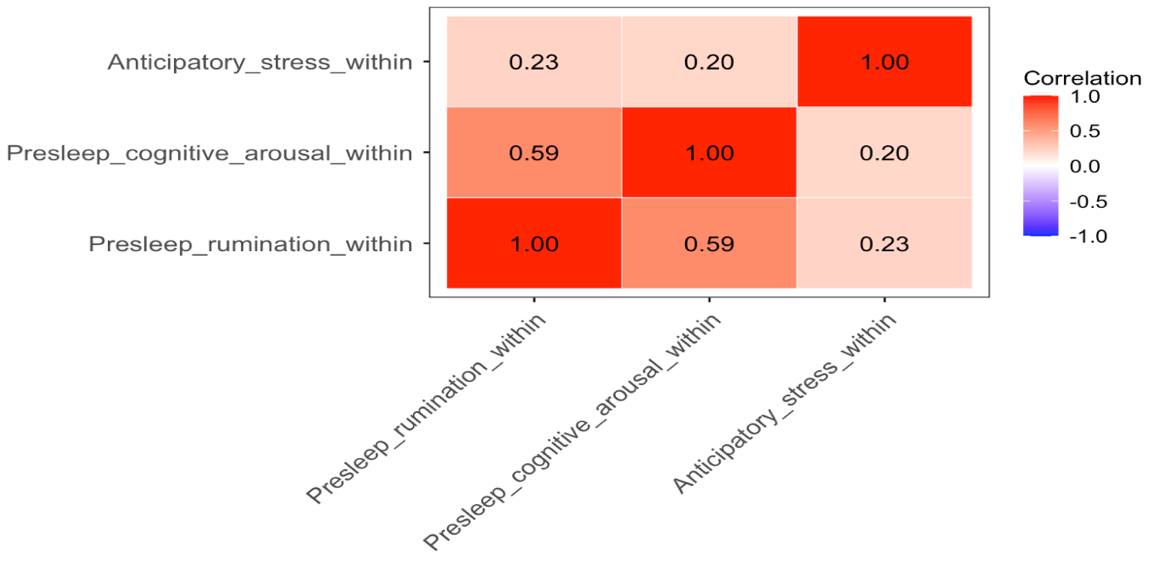


*Note.* Heatmap shows within-person Pearson correlations among pre-sleep cognitive arousal, pre-sleep rumination, and anticipatory stress.

**Figure S3**

*Associations between baseline trait measures and daily mean levels of between-person cognitive processes*


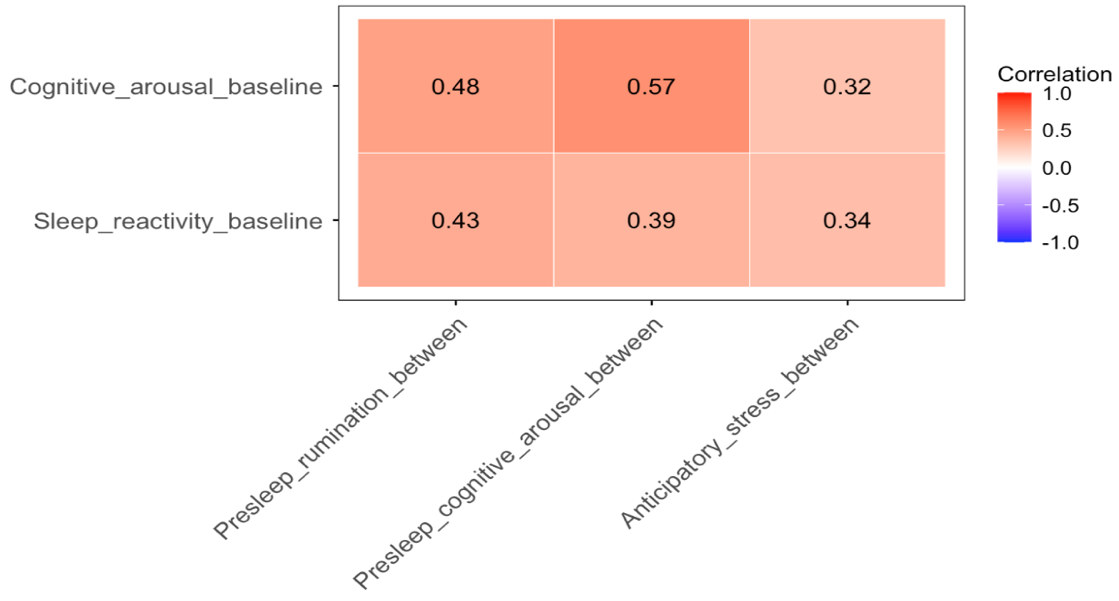


*Note*. Heatmap shows Pearson correlations between baseline trait measures (cognitive arousal and sleep reactivity) and daily between-person levels of pre-sleep cognitive arousal, pre-sleep rumination, and anticipatory stress (person-level means across the assessment period).

**Table S2**

*Associations between pre-sleep cognitive processes and sleep outcomes with covariates*

|  |  | **Fixed effects** | | | |
| --- | --- | --- | --- | --- | --- |
| **Model** | **Predictor** | **Coefficient (*SE*)** | **95% CI** | ***p_FDR_*** | ***R²*** |
| **Sleep quality** |  |  |  |  |  |
| Pre-sleep cognitive arousal | Pre-sleep cognitive arousal (between) | -4.89 (1.61) | [-8.07, -1.72] | .029* | .020 |
|  | Pre-sleep cognitive arousal (within) | -0.85 (1.13) | [-3.05, 1.36] | .612 | .000 |
|  | Day | -0.24 (0.33) | [-0.89, 0.42] | .612 | .000 |
|  | Study site | -1.44 (2.97) | [-7.29, 4.40] | .659 | .001 |
|  | Age | 0.56 (0.85) | [-1.11, 2.24] | .612 | .001 |
|  | Female | 9.02 (5.61) | [-2.02, 20.06] | .275 | .006 |
|  | Male | 11.68 (6.31) | [-0.72, 24.09] | .275 | .008 |
| Pre-sleep rumination | Pre-sleep rumination (between) | -1.40 (0.52) | [-2.44, -0.37] | .058 | .016 |
|  | Pre-sleep rumination (within) | 0.10 (0.41) | [-0.69, 0.89] | .802 | .000 |
|  | Day | -2.10 (3.36) | [-8.68, 4.47] | .612 | .000 |
|  | Study site | -20.75 (29.49) | [-78.76, 37.26] | .612 | .001 |
|  | Age | 5.07 (8.55) | [-11.76, 21.91] | .612 | .001 |
|  | Female | 97.06 (56.22) | [-13.52, 207.64] | .275 | .007 |
|  | Male | 122.45 (63.11) | [-1.69, 246.60] | .275 | .009 |
| Anticipatory stress | Anticipatory stress (between) | -2.00 (0.61) | [-3.20, -0.79] | .027* | .023 |
|  | Anticipatory stress (within) | -0.25 (0.32) | [-0.88, 0.37] | .612 | .001 |
|  | Day | -2.09 (3.35) | [-8.63, 4.46] | .612 | .000 |
|  | Study site | -24.72 (28.84) | [-81.45, 32.02] | .612 | .002 |
|  | Age | 8.41 (8.54) | [-8.38, 25.20] | .612 | .002 |
|  | Female | 87.75 (55.80) | [-22.01, 197.51] | .275 | .006 |
|  | Male | 100.67 (63.35) | [-23.94, 225.29] | .275 | .006 |
| **Sleep duration** |  |  |  |  |  |
| Pre-sleep cognitive arousal | Pre-sleep cognitive arousal (between) | -2.54 (1.01) | [-4.53, -0.55] | .046* | .014 |
|  | Pre-sleep cognitive arousal (within) | -1.31 (0.73) | [-2.73, 0.11] | .216 | .003 |
|  | Day | 0.23 (0.22) | [-0.19, 0.66] | .421 | .001 |
|  | Study site | -5.21 (1.86) | [-8.88, -1.54] | .026* | .017 |
|  | Age | -0.23 (0.54) | [-1.28, 0.82] | .703 | .000 |
|  | Female | -2.27 (3.54) | [-9.23, 4.68] | .644 | .001 |
|  | Male | -5.37 (3.97) | [-13.17, 2.44] | .344 | .004 |
| Pre-sleep rumination | Pre-sleep rumination (between) | -0.91 (0.33) | [-1.56, -0.26] | .026* | .017 |
|  | Pre-sleep rumination (within) | -0.35 (0.26) | [-0.86, 0.16] | .344 | .001 |
|  | Day | 2.40 (2.16) | [-1.83, 6.62] | .421 | .001 |
|  | Study site | -53.20 (18.43) | [-89.45, -16.94] | .026* | .018 |
|  | Age | -2.57 (5.35) | [-13.09, 7.95] | .698 | .001 |
|  | Female | -19.53 (35.29) | [-88.94, 49.88] | .677 | .001 |
|  | Male | -53.22 (39.57) | [-131.06, 24.61] | .344 | .004 |
| Anticipatory stress | Anticipatory stress (between) | -1.27 (0.38) | [-2.02, -0.53] | .021* | .024 |
|  | Anticipatory stress (within) | 0.14 (0.21) | [-0.26, 0.55] | .631 | .000 |
|  | Day | 2.54 (2.16) | [-1.67, 6.77] | .417 | .001 |
|  | Study site | -56.16 (17.92) | [-91.42, -20.90] | .022* | .021 |
|  | Age | -0.50 (5.30) | [-10.93, 9.94] | .925 | .000 |
|  | Female | -25.60 (34.83) | [-94.12, 42.92] | .631 | .001 |
|  | Male | -66.72 (39.50) | [-144.41, 10.97] | .244 | .006 |
| **Sleep onset latency** |  |  |  |  |  |
| Pre-sleep cognitive arousal | Pre-sleep cognitive arousal (between) | 0.54 (0.79) | [-1.01, 2.09] | .935 | .001 |
|  | Pre-sleep cognitive arousal (within) | 0.46 (0.49) | [-0.49, 1.42] | .935 | .001 |
|  | Day | -0.01 (0.14) | [-0.30, 0.27] | .935 | .000 |
|  | Study site | -1.08 (1.46) | [-3.95, 1.78] | .935 | .001 |
|  | Age | -0.15 (0.42) | [-0.98, 0.67] | .935 | .000 |
|  | Female | 1.59 (2.75) | [-3.83, 7.01] | .935 | .001 |
|  | Male | -1.43 (3.09) | [-7.52, 4.66] | .935 | .001 |
| Pre-sleep rumination | Pre-sleep rumination (between) | 0.26 (0.26) | [-0.24, 0.76] | .935 | .003 |
|  | Pre-sleep rumination (within) | 0.01 (0.17) | [-0.33, 0.36] | .935 | .000 |
|  | Day | -0.26 (1.45) | [-3.10, 2.58] | .935 | .000 |
|  | Study site | -11.29 (14.41) | [-39.63, 17.05] | .935 | .002 |
|  | Age | -1.44 (4.18) | [-9.67, 6.79] | .935 | .000 |
|  | Female | 15.39 (27.48) | [-38.66, 69.45] | .935 | .001 |
|  | Male | -13.54 (30.85) | [-74.22, 47.14] | .935 | .001 |
| Anticipatory stress | Anticipatory stress (between) | -0.23 (0.30) | [-0.82, 0.36] | .935 | .002 |
|  | Anticipatory stress (within) | -0.07 (0.14) | [-0.34, 0.20] | .935 | .000 |
|  | Day | -0.25 (1.44) | [-3.09, 2.58] | .935 | .000 |
|  | Study site | -6.81 (14.28) | [-34.89, 21.27] | .935 | .001 |
|  | Age | -1.08 (4.23) | [-9.40, 7.24] | .935 | .000 |
|  | Female | 13.84 (27.64) | [-40.52, 68.20] | .935 | .001 |
|  | Male | -21.59 (31.38) | [-83.31, 40.12] | .935 | .001 |

*Note*. Between-person associations were modeled using person-level means, and within-person associations were modeled using cluster-mean centering. *SE* = Standard error of the coefficient; 95% CI = Lower (2.5%) and upper limit (97.5%) of the confidence interval with 95%; *p_FDR_* = Benjamini–Hochberg false discovery rate procedure corrected p-value; *R²* = Proportion of variance in sleep quality uniquely explained by each fixed effect.

**p_FDR_* < .05, ***p_FDR_* < .01, ****p_FDR_* < .001.
